# Continuous intracisternal nimodipine administration as rescue therapy for refractory vasospasm in patients with aneurysmal subarachnoid haemorrhage

**DOI:** 10.1007/s00701-025-06702-5

**Published:** 2025-11-04

**Authors:** Mandy D. Müller, Katharina Janosovits, David Bervini, Pasquale Mordasini, Tomas Dobrocky, Eike I. Piechowiak, Joerg C. Schefold, Michael Murek, Johannes Goldberg, Philippe Schucht, Andreas Raabe, Werner J. Z’Graggen

**Affiliations:** 1 Department of Neurosurgery, Inselspital, Bern University Hospital, University of Bern, Bern, Switzerland; 2 Institute of Diagnostic and Interventional Neuroradiologiy, Center for Imaging and minimally invasive Therapies, Kantonsspital Aarau (KSA), Aarau, Switzerland; 3 Department of Neuroradiology, Inselspital, Bern University Hospital, University of Bern, Bern, Switzerland; 4 Department of Intensive Care Medicine, Inselspital, Bern University Hospital, University of Bern, Bern, Switzerland; 5 Department of Neurology, Bern University Hospital and University of Bern, Bern, Switzerland

**Keywords:** Delayed cerebral ischaemia, Cerebral vasospasm, Functional outcome, Cerebral perfusion, Cerebral infarction

## Abstract

**Purpose:**

Delayed cerebral ischaemia (DCI) and cerebral vasospasm (CVS) remain major causes of poor outcome in survivors of aneurysmal subarachnoid haemorrhage (aSAH). We aimed to investigate the safety and efficacy of intracisternal administration of nimodipine in patients suffering from symptomatic CVS refractory to treatment with induced hypertension and endovascular vasodilator therapy.

**Methods:**

We performed a single-centre, retrospective, observational study including all patients diagnosed with refractory CVS after aSAH treated at our tertiary centre between January 2018 and December 2021 who received continuous intracisternal nimodipine. For nimodipine administration, a catheter was inserted in the optico-carotid cistern via supraorbital craniotomy. Our primary outcome was functional independence measured by the modified Rankin Scale (mRS) at 6 months. Secondary outcomes included treatment related complications and neurological outcome.

**Results:**

We included 15 patients in total. Clinical outcome measured by the mRS at 6 months was good with 93.3% of patients showing mRS ≤ 1 (median mRS 1; range 1–4) Eight patients (53%) developed a new CVS-related neurological deficit during intrathecal nimodipine treatment and additionally received bolus intra-arterial nimodipine. Two patients (13%) developed acute subdural/epidural hematoma postoperatively, which was treated surgically in one patient. In two patients (13%), accidental dislocation of the intrathecal catheter occurred, which warranted re-operation.

**Conclusion:**

Continuous intracisternal administration of nimodipine may be a viable rescue therapy option for patients with refractory CVS but is associated with an increased risk of treatment related complications.

## Introduction

Aneurysmal subarachnoid haemorrhage (aSAH) occurs in approximately 9 people per 100.000 per year [1]. To this day morbidity and mortality associated with aSAH remains high [1]. Only approximately 50% of those who experience aSAH survive [2–5]. Due to the high morbidity and the long-term sequelae associated with this disease, a substantial part of patients who survive aSAH will never return to work or lead independent lives [6–9]. In patients who survive the initial bleeding, the main reasons for delayed worsening of neurological outcome and/or death are delayed cerebral ischaemia (DCI) and cerebral vasospasm (CVS) [10, 11]. Clinically symptomatic CVS are common and occur in approximately 20–25% of SAH patients [12] of whom 20–40% develop DCI-related cerebral ischaemia [13–15].

Nimodipine is commonly used for DCI prevention as several studies have reported a lowered incidence of cerebral infarction and poor outcome after aSAH associated with oral nimodipine administration [10, 16–19]. Interestingly, nimodipine did not lower the incidence of angiographic vasospasm but it reduced the occurrence of death or severe disability significantly when administered orally every four hours for 21 days or until CVS occurred [18]. Commonly used rescue treatments for symptomatic CVS include induced hypertension and in refractory CVS, intra-arterial administration of vasodilators or angioplasty. However, the efficacy of these treatments is not proven, their effects are temporary at best and potentially severe complications may occur [20–23].

In the absence of alternatives, many centres still advocate induced hypertension and interventional treatment strategies such as repeated bolus injections of nimodipine or balloon angioplasty as rescue therapies despite the lack of high-level scientific evidence.

Intra-arterial bolus injection of nimodipine has a relatively short duration of action and may cause side effects such as systemic hypotension [22]. Furthermore, due to the short action time, repeated interventions with intra-arterial bolus injections may be necessary for the treatment of recurrent, severe CVS, which in turn increases the risk for intervention-associated complications such as vessel dissection, thromboembolic events or infection.

Alternatively, intrathecal administration of vasodilating drugs or fibrinolytic agents have been proposed [24, 25]. The potential advantages of these treatments include high concentrations of vasodilating substances surrounding the arteries affected by CVS, the possibility of continuous or sustained-release formulations and potentially less systemic adverse effects such as hypotension [25–27]. However to date, results from studies investigating the effects of intrathecal administration of nimodipine have been conflicting [27–29].

We performed a retrospective analysis of patients treated with intracisternal nimodipine at our centre between January 2018 and December 2021 for severe, therapy refractory CVS after aSAH. The main aim of our study was to investigate the safety and efficacy of intracisternal administration of nimodipine.

## Methods

We included all patients with refractory CVS after aneurysmal SAH who presented at our tertiary centre between January 2018 and December 2021. According to our institutional standard operating procedures, the following patients were considered for intracisternal nimodipine treatment:

- Persisting new neurological deficit attributed to CVS after initiation of maximal first-line therapy consisting of induced hypertension with norepinephrine (MAP + 10–20 mmHg) and normo- to mild hypervolemia or
- Persisting new neurological deficit attributed to CVS after at least one rescue intervention with intra-arterial bolus administration of nimodipine had been performed previously or
- Diffuse angiographic vasospasm observed in the first angiography, which responded only insufficiently to intra-arterial bolus nimodipine.

CVS was defined as any new neurological deficit and/or acute GCS decline of ≥ 2 points after other potential causes (i.e. infection, over-sedation, seizure, hydrocephalus) had been excluded [30].

Patient demographics including severity of aSAH (Fischer, WFNS, BNI, Hunt and Hess Grade) were recorded at baseline. Patients with documented refusal of participation in research projects were excluded from the analysis. Approval for this study was obtained from the local ethics committee on human research (Kantonale Ethikkommission, KEK, Bern, Switzerland, BASEC-Nr: 2022–00310) and the study conformed to the Declaration of Helsinki.

**Fig. 1:**
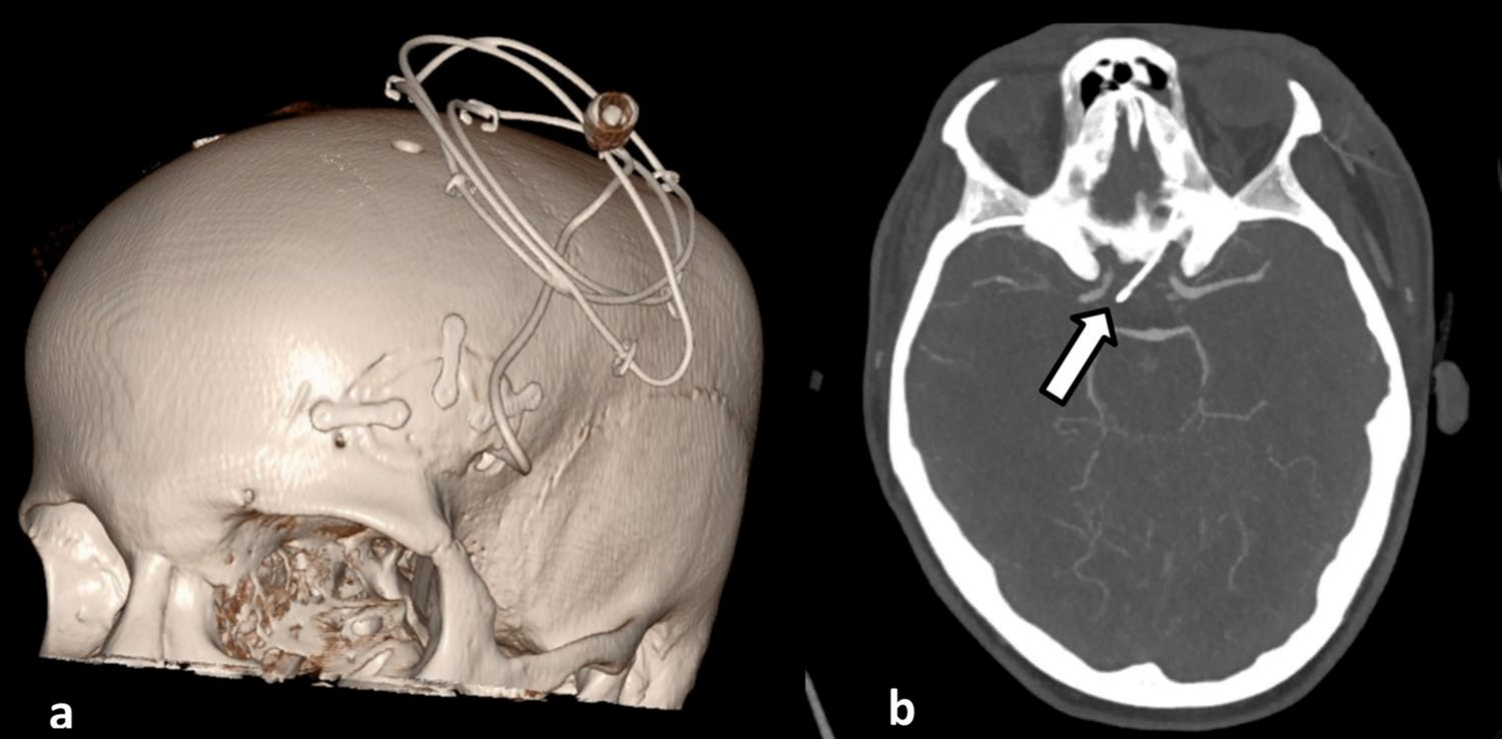
**a**: 3D reconstructed CT of a patient with intracisternal nimodipine catheter, which was inserted through a lateral supraorbital mini-craniotomy. **b: **axial CT showing the intracranial position of the nimodipine catheter in the optico-carotid cistern (white arrow)

For continuous intrathecal nimodipine administration, a catheter was inserted in the optico-carotid cistern unilaterally via lateral supraorbital craniotomy performed through an eyebrow incision (Fig. 1a and b). The catheter was placed on the side with more pronounced vasospasm on cerebral imaging. If both sides were affected equally, the non-dominant, right side was chosen for catheter insertion. After dural opening, the basal cisterns were fenestrated through a subfrontal approach and the catheter (Spiegelberg external Silverline® lumbar drainage catheter, Spiegelberg GmbH & Co. KG, Hamburg, Germany) was inserted under visual control. Nimodipine was infused continuously using a perfusor pump. Standard infusion rate for nimodipine was 0.25 mg/hour. Postoperatively, patients were extubated in a timely manner and neurological function was assessed through hourly neurological examination, assessment of Glascow Coma Scale (GCS) as well as Richmond Agitation and Sedation Scale (RASS). Standard management including bedrest, induced hypertension, normo- to mild hypervolemia, as well as thrombosis prophylaxis with heparin was continued. Patients who developed symptomatic hydrocephalus received an external ventricular drain. In those cases, intracranial pressure (ICP) was monitored and documented continuously. The volume of cerebrospinal fluid drained per day was recorded. Patients without symptomatic hydrocephalus did not receive an external ventricular drain.

Nimodipine was tapered off when patients were clinically stable for at least 48–72 h. The infusion rate was reduced to 0.125 mg/hour. If patients remained clinically stable, intrathecal nimodipine application was stopped 48 h thereafter. All patients received overlapping intravenous or oral nimodipine to prevent rebound CVS (60 mg oral nimodipine every 4 h as soon as an intrathecal dosage of 0.125 mg/h nimodipine was reached).

In all patients, the number of days of nimodipine administration, cumulative dosage of nimodipine infused, as well as the nimodipine infusion rates were recorded. The need for vasoactive drugs and their dosage was also documented. Rebound CVS defined as any new focal neurological deficit occurring during the weaning of continuous intrathecal or after cessation thereof were recorded.

As a standard of care, all patients received cerebral imaging (MRI or CT) 24 h after securing the ruptured aneurysm (MRI 1 or CT 1) as well as cerebral MRI after cessation of intrathecal nimodipine treatment (MRI 2). DCI-related cerebral ischaemia was defined as any new cerebral ischaemia identified on MRI 2, which had not been present in MRI 1 or CT 1. The total volume of new cerebral ischaemia seen on diffusion weighted imaging was calculated using Brainlab smart brush software (Brainlab AG, Munich, Germany).

The primary outcome of our study was functional independence measured by the modified Rankin Scale (mRS) at 6 months. Secondary outcomes included functional independence (measured by mRS) at 3 months and neurological outcome measured by the NIH Stroke Scale (NIHSS) at 3 and 6 months. Patients were followed clinically at 3 and 6 months. mRS was assessed using structured interviews by a member (resident or attending) of the Neurosurgical Department, who had not been directly involved in the care of the patient during nimodipine administration. For patients, who were dependent on continuing care (mRS ≥ 4), we used structured interviews with the patient’s care provider to assess mRS. We also recorded the occurrence of treatment associated complications including cerebral thromboembolism, intracranial haemorrhage, dislocation of intrathecal catheters, and infection including systemic and intracranial infections.

Additional secondary outcomes include descriptive analyses of data on DCI-related cerebral ischaemia volume, intracranial pressure (ICP) changes, duration and dosage of nimodipine administration.

## Results

We included 15 participants suffering from symptomatic CVS refractory to treatment with induced hypertension and endovascular vasodilator therapy in this analysis. Table 1 shows baseline characteristics of our study population.

**Table 1:** Baseline characteristics

**Age**, mean, range	53 yrs (27-65yrs)
**Female**, n (%)	9 (56%)
**Aneurysma location** n (%)	
** ACA**	1 (6%)
** Acom**	7 (44%)
** ICA**	1 (6%)
** ChA**	1 (6%)
** MCA**	3 (19%)
** Pcom**	1 (6%)
** PICA**	1 (6%)
**Treatment modality** n (%)	
** Coiling**	14 (94%)
** Clipping**	1 (6%)
**WFNS**, median (IQR)	1 (1–5)
**hWFNS**, median (IQR)	1 (1–4)
**Fisher**, median (IQR)	3 (3–4)
**BNI**, median (IQR)	4 (3–5)

Baseline characteristics of patients included in the analysis. *ACA* anterior cerebral artery, *Acom* anterior communicating artery, *ICA* internal cerebral artery, *ChA* anterior choroidal artery, *MCA* middle cerebral artery, *Pcom* posterior communicating artery, *PICA* posterior inferior cerebellar artery, *WFNS* World Federation of Neurosurgical Societies Score, *hWFNS* herniation WFNS score adapted from Fung C et al. Neurosurg. 2016 Feb;124(2):299–304. *BNI* Barrow Neurological Institute Grading Scale, *IQR* Interquartile Range

All patients included in this analysis were admitted to the neuro-intermediate care unit or intensive care unit. The ruptured aneurysm was treated within 24 h. The majority of patients received endovascular treatment of their ruptured aneurysm (Table 1). As a standard of care at our centre, all patients received CT or MRI after aneurysm treatment irrespective of treatment modality (endovascular or surgical) for evaluation of treatment associated complications e.g. ischemia or haemorrhage.

### Outcome and duration of nimodipine administration

**Fig. 2:**
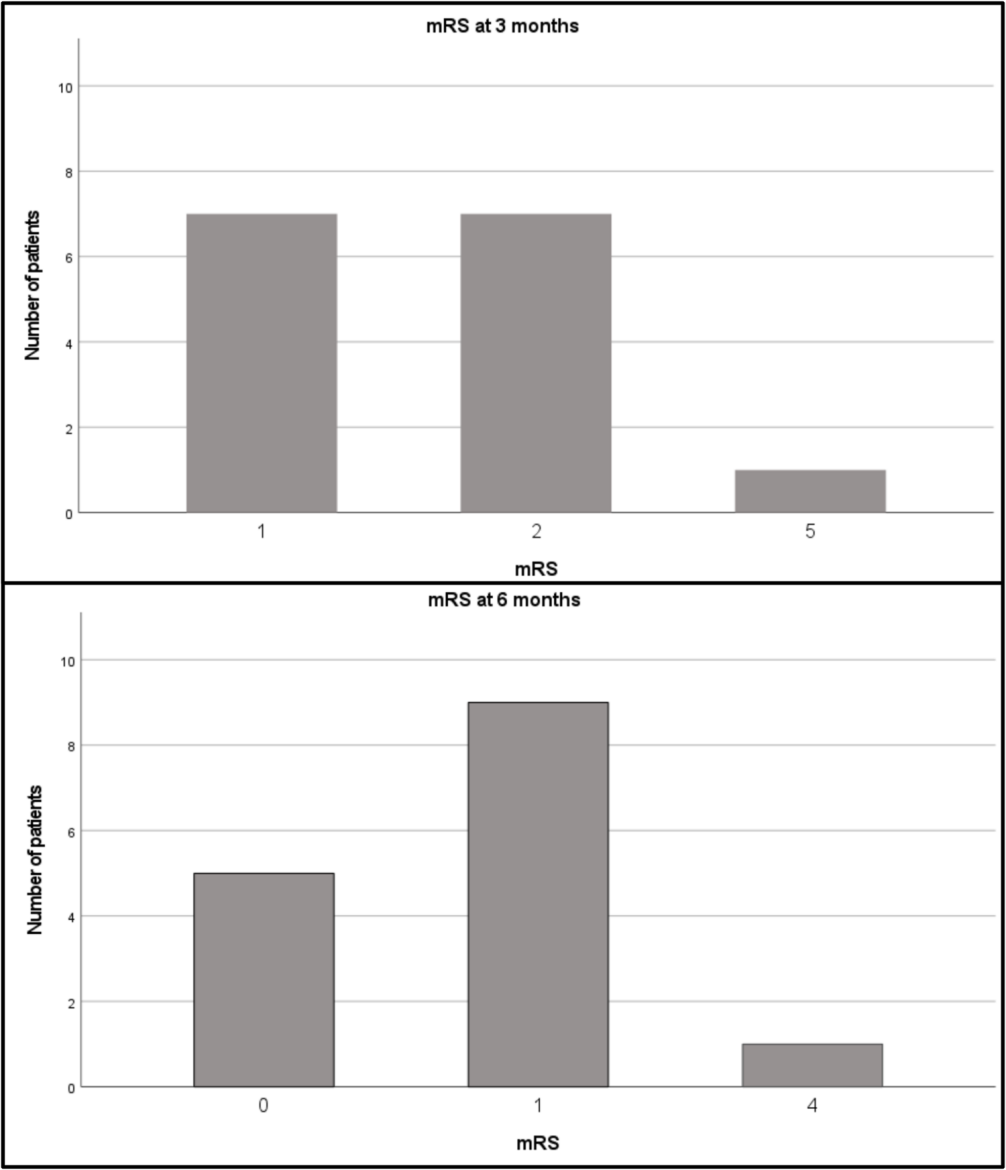
Bar charts depicting the distribution of patients according to their modified Rankin Scale (mRS) scores at 3 and 6 months. The x-axis represents the mRS scores, while the y-axis represents the number of patients

Functional independence measured by the modified Rankin Scale (mRS) at 6 months was good with a median mRS of 1 (range 0–4; Fig. 2). Neurological outcome after 6 months was also good with a median NIHSS 1 (range 0–7). Overall, 93.3% of patients showed mRS ≤ 1 after 6 months. After 3 months, median mRS was 2 (range 1–5) and median NIHSS was 1 (range 0–22). One patient (6.7%) died two years after the aSAH. However, the death was not related to the initial haemorrhage.

The mean interval between ictus and implantation of the intrathecal catheter was 8.3 days (range 4–12 days). The mean cumulative infused nimodipine dosage was 87.3 mg (range 17.7–446.7 mg). On average, the catheter remained in place for 8 days (range 4–18 days). Rebound CVS after removal of the catheter occurred in three patients (20%; Table 2).

**Table 2:** Treatment related as well as not procedure related complications

**Procedure related** n (%)	
** CNS infections**	1 (6.7%)
** Haemorrhage**	
** Subdural/epidural**	2 (13%)
** Surgical treatment necessary**	1 (6.7%)
** Asymptomatic subdural hygroma** n (%)	5 (33.3%)
** Dislocation of catheter** n (%)	2 (13%)
** Rebound CVS** n (%)	3 (20%)
** Other** n (%)	1 (6.7%)^†^
**Not procedure related** n (%)	
** Cerebral thromboembolism**	2 (13%)*
** Systemic Infections**	5 (33.3%)

Outlining all complications in our cohort including procedure associated complications

*occurring during repeat intra-arterial bolus administration of nimodipine, complete endovascular revascularisation was possible in both cases, ^†^surgical treatment of CSF leak

Overall, 13 (87%) patients were monitored with an external ventricular drain (EVD). Intracranial pressure was well controlled before and during treatment with intrathecal nimodipine. In one patient however, intrathecal nimodipine administration had to be halted due to an increase in intracranial pressure, which could not be controlled with conservative means. Due to continuing severe CVS, this patient was treated with continuous intra-arterial nimodipine after cessation of intrathecal nimodipine administration.

Eight patients (53%) treated with intrathecal nimodipine developed new symptomatic CVS during the intrathecal administration of nimodipine and additionally received bolus intra-arterial spasmolysis.

The mean volume of new ischaemic DCI-related lesions after completion of treatment was 6.7 ml (range 0–44.4 ml). Five (33.3%) patients did not show any new ischaemic lesions.

### Complications of intracisternal nimodipine administration

In two (13%) patients, dislocation of the intracisternal catheter occurred postoperatively due to accidental traction on the catheter, which warranted re-operation for correct placement of the catheter. Furthermore, two (13%) patients developed acute epi-/subdural hematoma, which had to be treated surgically in one (6.7%) case (Table 2). One (6.7%) patient developed meningitis which was treated with antibiotics. Moreover, five (33.3%) patients developed asymptomatic subdural hygroma, most likely due to the accumulation of the infused nimodipine. In all cases, the subdural hygroma resolved without treatment after cessation of nimodipine application. One (6.7%) patient developed a cerebrospinal fluid leak after the removal of the intrathecal catheter and had to undergo revision surgery. Two (13%) patients who received intra-arterial bolus spasmolysis in addition to the intrathecal nimodipine catheter developed cerebral thromboembolism after intra-arterial spasmolysis (Table 2).

### Systemic complications

Five (33.3%) patients developed systemic infections (urinary tract infections, pneumonia) not related to the catheter treatment (Table 2).

## Discussion

This retrospective study indicates that continuous intracisternal administration of nimodipine may be a viable add-on rescue therapy option for patients with refractory CVS but is associated with an increased risk of treatment related complications.

Commonly used rescue treatments for symptomatic CVS include induced hypertension, and in refractory CVS, angioplasty or intra-arterial administration of vasodilators. However, the efficacy of these interventions is still not established, their effects are temporary at best, and adverse effects are common [20–22].

Therefore, the optimal management of refractory CVS after aSAH remains challenging. Previous case series showed beneficial effects of repeated bolus injections of nimodipine in the affected cerebral artery and/or balloon angioplasty but a randomized controlled trial had to be stopped prematurely and failed to prove efficacy [21]. Both, bolus injection of nimodipine and balloon angioplasty are not without risks and potentially severe complications may arise as it was also the case in our series with two thromboembolic events during bolus-nimodipine administration.

As an alternative, intrathecal administration of nimodipine has been investigated in several previous studies. The NEWTON phase 1/2 trial was a randomised, open-label, dose-escalation study investigating the effects of sustained-release intraventricular formulation of nimodipine for the prevention of DCI. The study showed promising results [29]. However, the following phase 3 trial showed no difference in favourable clinical outcome in patients treated with intraventricular nimodipine compared to those treated with placebo [28].

Roelz and colleagues developed an alternative method to deliver intrathecal nimodipine and urokinase for the prevention of DCI in patients with aSAH. Their approach involves stereotactic placement of a catheter into the basal cisterns, followed by continuous cisternal lavage with urokinase and nimodipine (STX-VCS). A study comparing the effects of STX-VCS to a matched cohort found a significant reduction in the occurrence of DCI and mortality as well as significantly higher rate of favourable functional outcome compared to the matched cohort [27]. A randomised trial evaluating the effects of this method is currently ongoing [31]. As in these previous studies [27, 29], intrathecal nimodipine was tolerated well in our cohort and had the advantage that the patients were awake and could be followed clinically. Additionally, our surgical approach did not require a stereotactic frame for placement of the catheters. We hypothesized that cisternal administration of nimodipine directly into the optico-carotid cistern may offer more targeted drug delivery to the affected arteries, potentially overcoming limitations of intraventricular nimodipine administration, where drug distribution may be less direct and subject to CSF flow dynamics. However, intracisternal therapy delivered through continuous infusion in the optico-carotid cistern appears to have limited efficacy, as measured by the rather high number (8/15; 53%) of additional bolus intra-arterial nimodipine treatments required during the period of intrathecal therapy. Furthermore, the rate of complications associated with intrathecal catheter placement was high: two patients (13%) developed an acute epi- or subdural haematoma, which warranted emergent surgical evacuation in one patient. In two patients (13%), the catheter was accidentally dislocated during hospitalisation, which required re-operation for correct placement of the catheter.

Twenty percent of the patients undergoing intrathecal nimodipine treatment developed rebound CVS after cessation of the intrathecal drug administration. These rebound CVS were observed despite stepwise weaning of nimodipine and during oral replacement. Typically, rebound CVS became symptomatic within 24 to 36 h after the start of the oral therapy. Three of these patients needed intra-arterial spasmolysis with bolus nimodipine. None required repeat interventions.

The major limitations of this study are the retrospective design, the limited number of participants, and the single-center approach. Therefore, our findings have to be interpreted with caution and prospective studies with larger sample sizes are warranted to investigate the exact safety and efficacy profiles of continuous intrathecal nimodipine administration.

Our results are descriptive and, though promising, do not allow conclusions on the efficacy of intracisternal nimodipine since no control group was available and the rate of additional bolus intra-arterial nimodipine administration was high. Nevertheless, in patients with DCI, morbidity and mortality is high and treatment options are limited [20, 32–34]. In a recent study, Suwatcharangkoon et al. reported poor outcomes (mRS 4–6) one year after aSAH in 62% of patients who did not respond to medical therapy for symptomatic CVS [35]. We studied a comparable cohort as all included patients had developed refractory CVS before inclusion in our study and initiation of continuous intracisternal  nimodipine. Nevertheless, clinical outcome in our study population was favourable (93.3% of patients had mRS ≤ 1 after 6 months) highlighting the promising effect of intracisternal nimodipine even if high-quality evidence supporting its use is still lacking and further prospective studies are needed.

## Conclusion

This retrospective study indicates that continuous intracisternal administration of nimodipine may be a viable add-on rescue therapy option for patients with refractory CVS but is associated with an increased risk of treatment related complications.

## Data Availability

The data supporting this article is available from the first author upon reasonable request.
